# Exposure to Toxicologically Relevant Inorganic Elements and Nicotine Content Variability in Electronic Nicotine Delivery System (ENDS) E‑Liquids

**DOI:** 10.1021/acs.chemrestox.6c00266

**Published:** 2026-08-31

**Authors:** Lara M. Catarino, Giovanna M. Iozzi, Cristiane B. Silva, Santos A.V. Neto, Graziele A. Reis, Silvana C. Jacob, Lisia M. G. dos Santos

**Affiliations:** † Inorganic Elements Sector, Department of Chemistry, National Institute for Quality Control in Health (INCQS), 37903 Oswaldo Cruz Foundation (Fiocruz), Rio de Janeiro, RJ 21040-900, Brazil; ‡ State University of Rio de Janeiro (UERJ), Maracanã, Rio de Janeiro, RJ 20550-013, Brazil

## Abstract

In recent years, electronic nicotine delivery systems (ENDS) have gained prominence as alternatives to conventional cigarettes, raising concerns due to the increasing number of lung injury cases associated with their use. These devices rely on e-liquids composed primarily of propylene glycol, glycerin, flavorings, and nicotine, which are heated to produce inhalable aerosols. In this context, the present study aimed to quantify nicotine concentrations and assess the presence of toxicologically relevant inorganic elements in e-liquids to evaluate potential inhalation-related risks. Nicotine was quantified using high-performance liquid chromatography with diode-array detection (HPLC–DAD), whereas inorganic contaminants were determined using inductively coupled plasma–mass spectrometry (ICP–MS). Nicotine concentrations ranged from nondetectable to 33.4 mg·mL^–1^, and significant discrepancies were identified in the labeling information. Arsenic (As), lead (Pb), nickel (Ni), chromium (Cr), and mercury (Hg) were detected at variable concentrations; notably, the estimated daily exposures for Cr and Hg exceeded the permitted daily exposure (PDE) limits established by the International Council for Harmonization (ICH). Overall, these findings indicate that ENDS may be a significant source of toxic metal exposure, posing potential risks to human health.

## Graphical abstract

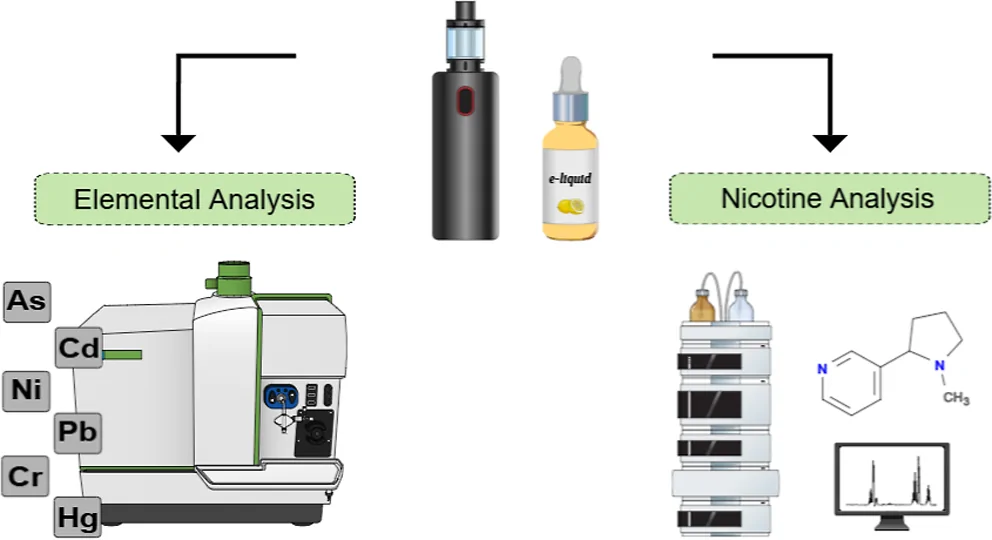

## Introduction

Recognized as a chronic disease resulting from nicotine dependence on tobacco-based products, smoking remains the leading preventable cause of morbidity and mortality worldwide, accounting for more than eight million deaths annually.^1^ As awareness of the health risks associated with conventional cigarettes has grown, the tobacco industry has diversified its portfolio to attract new consumers, with electronic nicotine delivery systems (ENDS) becoming a major focus.^2^

First introduced to the market in 2003 under the premise of being less harmful to health, ENDS were commercialized as an alternative to traditional cigarettes.^3^ These devices generally comprise a battery-powered electronic system and a cartridge containing liquids, also known as e-juices or e-liquids, formulated from propylene glycol, glycerin, flavorings, and nicotine at varying concentrations. During use, the e-liquid is heated and aerosolized, producing vapors that are inhaled by the user.^4^

In countries where their commercialization is permitted, such as the United States, ENDS are currently the most widely used smoking product among adolescents and young adults.^5,6^ In Brazil, despite a regulatory ban in effect since 2009, approximately 0.64% of the population aged ≥15 years reported using ENDS, corresponding to nearly one million individuals. The highest prevalence was observed in the 15–24 year age group (2.38%), which represents almost 70% of current users.^7^

Given the growing number of reported cases of e-cigarette or vaping product use-associated lung injury (EVALI), several studies have examined the chemical composition of e-liquids and the aerosols they generate, with particular attention to toxicologically relevant contaminants.^8,9^ Elemental impurities (trace metals and nonmetals originating from manufacturing processes, device components, or natural sources) are of particular concern due to their potential health effects.^10^ In the absence of specific regulatory standards for ENDS, the ICH Q3D (R1) guideline has been widely adopted as a reference framework for assessing inhalation exposure, as it defines permitted daily exposure (PDE) limits for elemental impurities. Against this backdrop, this study aimed to determine nicotine concentrations and quantify toxicologically relevant inorganic elements of inhalation concern in e-liquids, such As, Cd, Pb, Ni, Cr, and Hg, to evaluate the potential risks associated with human exposure.^10^

## Methods

### Sample Acquisition

The samples were obtained through a partnership with federal regulatory agencies that provided 22 seized e-liquid samples for analysis. Among these, 16 samples (RC01-RC16) labeled as nicotine-free were from the same brand but varied by flavor, whereas the remaining six samples (VPJ1–VPJ6) contained nicotine and represented different brands and flavors. Analyses of nicotine and inorganic elements were conducted at the Department of Chemistry and Inorganic Elements Sector of the National Institute for Quality Control in Health (INCQS), Oswaldo Cruz Foundation (Fiocruz).

### Sample Preparation

For inorganic contaminant analysis, 0.3 g of each sample was weighed and digested with 1.1 mL of 65% (w/v) HNO_3_ (Supelco), 0.8 mL of 30% (w/w) H_2_O_2_ (Sigma-Aldrich), and 6.1 mL of deionized water. Digestion was performed using a closed-vessel microwave system (Titan MPS; PerkinElmer). The digestion program consisted of two heating steps: samples were heated to 100 °C with a 2 min ramp and held for 5 min, followed by heating to 160 °C with a 5 min ramp and held for 20 min at 90% microwave power (maximum pressure of 50 bar). The vessels were allowed to cool to approximately 50 °C and subsequently to room temperature before being opened. The digested solutions were transferred to 15 mL polypropylene tubes and diluted with deionized water prior to ICP–MS analysis.

For nicotine quantification, 1.0 mL of each sample was transferred to a 25.0 mL volumetric flask and diluted to volume with a 50% (v/v) isopropanol/water solution. The samples were subsequently analyzed by direct injection into the chromatographic system. Method blanks and independently prepared matrix-spiked quality control (QC) samples were included in each analytical batch for both analytical methods to monitor potential contamination and verify method performance.

### Analysis of Potentially Toxic Inorganic Elements in e-Liquids

The concentrations of inorganic elements were determined using inductively coupled plasma mass spectrometry (ICP–MS; NexION 300D, PerkinElmer) equipped with a Meinhard concentric nebulizer, a glass cyclonic spray chamber, and nickel sampler, skimmer, and hyper-skimmer cones. Argon (Ar, 99.996%) was used as both the plasma and carrier gas. Arsenic (^75^As), cadmium (^111^Cd), lead (^208^Pb), nickel (^60^Ni) and mercury (^202^Hg) were measured in standard mode, whereas chromium (^52^Cr) was quantified using a collision cell with helium gas (He, 99.999%) to reduce spectral interferences and improve analytical selectivity. Instrument operating conditions are summarized in Table S1. For each analytical batch, a new calibration curve consisting of six levels across the analytical working range was prepared to a final volume of 15 mL in polypropylene tubes by serial dilution of single-element standard solutions (Supelco, 1000 mg·L^–1^) with 1% (v/v) HNO_3_. The calibration standards were analyzed sequentially from the lowest to the highest concentration, and only curves with a coefficient of determination (R^2^) ≥ 0.990 were accepted for sample quantification. Rhodium (Rh) from a single-element standard (1000 mg·L^–1^, Supelco) was used as an internal standard at a concentration of 10 μg·L^–1^ and added to all calibration standards, blanks and digested samples. To minimize potential carryover, a 1% (v/v) HNO_3_ blank was analyzed after each calibration curve, and the sample introduction system was rinsed with an HNO_3_/Au solution after every three sample analyses. The ICP–MS method was validated before application to sample analysis; details of the validation procedure and analytical performance are provided in the Validation section.

### Analysis of Nicotine Content in e-Liquids

Nicotine concentrations were determined using high-performance liquid chromatography coupled with a UV–vis diode array detector (HPLC-DAD; Class-VP, Shimadzu). Separation was achieved using an ACE C8 column (250 × 4.6 mm, particle size 5 μm, pore size 100 Å). The mobile phase comprised phase A (triethylamine formate buffer, pH 5) and phase B (acetonitrile). Elution was carried out using the following gradient (time in minutes, % B): 0–5 min, 5% B; 5–15 min, 5–95% B; 15–20 min, 95% B; and 20–35 min, 5% B. The flow rate was 0.7 mL·min^–1^ and the injection volume was 15 μL. The detection was performed at 254 nm. Calibration curves were prepared using five concentration levels ranging from 10 to 1000 mg·L^–1^ from the European Pharmacopoeia nicotine ditartrate reference standard (CAS No. 65-31-6), prepared in a 50% (v/v) isopropanol/water solution. All calibration curves exhibited R^2^ ≥ 0.990. The HPLC-DAD analysis was performed as a complementary analytical characterization of selected samples and was not subjected to full analytical validation.

### Validation

Method validation was conducted in accordance with the guidelines of the National Institute of Metrology, Quality, and Technology (INMETRO) and ISO 17025.^11,12^ The evaluated performance characteristics included limits of detection (LOD) and quantification (LOQ), linearity, precision, accuracy, and matrix effect.

The LOD for each analyte was calculated using the equation LOD = 3.3 SD/*S*, where SD is the standard deviation of ten independent procedural blanks, and *S* is the slope of the corresponding calibration curve. The instrumental LOQ was established experimentally as the concentration corresponding to the first calibration level that fulfilled the predefined acceptance criteria for precision and accuracy. Method LOQs were calculated and expressed in μg·g^–1^ according to the sample mass and dilution factors applied during sample preparation.

Linearity was assessed using three independent calibration curves, each consisting of six concentration levels analyzed in triplicate. Linear regression analysis was performed, and the adequacy of the regression model was verified by assessing homoscedasticity, normality of residuals, and residual autocorrelation using the Brown–Forsythe, Ryan–Joiner, and Durbin–Watson tests, respectively. Regression ANOVA was used to evaluate the significance of the linear model, while the lack-of-fit test was applied to verify the absence of significant deviation from linearity. The analytical working ranges were established based on the statistical evaluation of the calibration data.

Precision was evaluated under repeatability and intermediate precision conditions. Repeatability was assessed by analyzing ten independently prepared matrix-spiked e-liquid samples at a midlevel concentration, while intermediate precision was assessed using ten independently prepared matrix-spiked e-liquid samples prepared and analyzed independently by two analysts on two different days. The results were expressed as %RSD, and Student’s *t*-test (95% confidence level) was used to verify the absence of statistically significant differences between analysts. Accuracy was determined through recovery experiments using ten independently prepared matrix-spiked e-liquid samples fortified at three concentration levels covering the working range (low, medium, and high). The experimental %RSD values and recovery results were compared with the concentration-dependent acceptance limits established by the INMETRO validation guideline.

The matrix effect was investigated by comparing calibration curves prepared in 1% (v/v) HNO_3_ with matrix-matched calibration curves prepared in representative digested e-liquid samples processed according to the analytical sample preparation procedure. The equivalence of the calibration curve slopes was assessed using Student’s *t*-test (95% confidence level).

### Statistical Analysis

Descriptive statistical analyses were performed using Microsoft Excel 2010 and included the mean, median, standard deviation (SD), Student’s *t*-test, and analysis of variance (ANOVA). To reduce variability in elemental concentration data, values were log_10_-transformed; however, normality tests indicated non-normal distributions of the data. Accordingly, nonparametric tests were applied: (1) Mann–Whitney U test for independent samples; (2) Kruskal–Wallis test for group comparisons; (3) Spearman’s correlation to assess monotonic relationships between variables; and (4) cluster analysis (CA) to identify similarity patterns among samples. All statistical analyses were performed using IBM SPSS Statistics version 29.0.

## Results and Discussion

### Validation

The evaluated performance characteristics met the acceptance criteria established by the INMETRO validation guidelines and ISO/IEC 17025, demonstrating the suitability of the proposed method for the determination of As, Cd, Pb, Ni, Cr and Hg in the analyzed samples (Table S2).

All the calibration curves showed satisfactory linearity across the validated working ranges, with coefficients of determination (R^2^) ranging from 0.9909 to 0.9999 for all analytes. The adequacy of the linear regression models was confirmed by the evaluation of regression assumptions, including homoscedasticity, normality of residuals, and residual independence. Regression ANOVA confirmed the statistical significance of the calibration models, and the lack-of-fit test indicated the absence of significant deviation from linearity, demonstrating that the selected working ranges were appropriate for quantitative analysis for all the analytes. The obtained LOQs were suitable for reliable quantification of the target analytes within the validated working ranges.

The precision results demonstrated satisfactory method performance under both repeatability and intermediate precision conditions. Repeatability (%RSD) ranged from 0.4 to 5.0%, while intermediate precision (%RSD) ranged from 0.9 to 6.7%. Student’s *t*-test showed no statistically significant differences between the results obtained by the two analysts (*p* > 0.05), confirming the reproducibility of the method under different analytical conditions. Accuracy, expressed as recovery, ranged from 79 to 114% and complied with the corresponding acceptance criteria at all evaluated concentration levels.

The evaluation of matrix effects showed no statistically significant differences between the slopes of calibration curves prepared in 1% (v/v) HNO_3_ and those prepared in representative digested e-liquid matrices (*p* > 0.05). These results indicate that the sample preparation procedure effectively minimized matrix-related interferences, supporting the use of external calibration with aqueous standards for the quantification of inorganic elements in e-liquid samples.

### Quantification of Potentially Toxic Inorganic Elements Present in e-Liquids

Arsenic concentrations in the analyzed e-liquids ranged from below the LOQ (<0.005 μg·g^–1^) to 0.068 μg·g^–1^. These values are consistent with those reported by Wang et al.,^13^ who observed As concentrations between 0.003 and 0.029 μg·g^–1^. All samples showed Cd concentrations below the LOQ, in agreement with previous reports for e-liquid samples.^8^ The absence of Cd may be associated with the use of synthetic nicotine, which is not derived from tobacco leaves and therefore lacks the inorganic contaminants commonly accumulated by plants from soil and environmental sources.^14^ Pb concentrations ranged from <0.005 to 0.051 μg·g^–1^, similar to those reported by Wang et al.^13^ (0.002–0.017 μg·g^–1^). Only three samples exhibited quantifiable levels of Ni, ranging from 0.053 to 0.149 μg·g^–1^. Previous investigations have reported variable Ni concentrations: Wang et al.^13^ found levels between 0.017 and 3.07 μg·g^–1^, while Gray et al.^15^ reported an average concentration of 0.063 μg·g^–1^ in four JUUL pod e-liquids. Higher Ni levels have been found in ENDS aerosols, likely due to metal leaching from heating coils and other device components.^16^ Among the elements analyzed, Cr exhibited the highest concentrations, ranging from <0.250 to 1.007 μg·g^–1^. These results are in line with those reported by Neu et al.,^17^ who found levels between 0.31 and 1.20 μg·g^–1^ in e-liquids from cigalike electronic cigarette cartridges. Although data on Hg in e-liquids remain scarce, nine samples showed quantifiable concentrations, ranging from 0.022 to 0.100 μg·g^–1^. Similar concentrations were reported by Wang et al.,^13^ with an average of 0.036 μg·g^–1^. The results for all analyzed samples are presented in Table 1.

**Table 1:** Concentrations of As, Cd, Pb, Ni, Cr, and Hg (μg·g^–1^) Found in the Analyzed e-Liquid Samples

sample Id	flavor	As (μg·g^–1^)	Cd (μg·g^–1^)	Pb (μg·g^–1^)	Ni (μg·g^–1^)	Cr (μg·g^–1^)	Hg (μg·g^–1^)
RC01	citrus mix	<0.005	<0.005	0.006	<0.025	0.293	0.033
RC02	pink peach	<0.005	<0.005	0.020	<0.025	0.363	<0.005
RC03	banana	<0.005	<0.005	0.026	<0.025	0.361	<0.005
RC04	strawberry	<0.005	<0.005	0.051	<0.025	<0.250	<0.005
RC05	watermelon	<0.005	<0.005	<0.005	<0.025	0.277	0.093
RC06	cherry	<0.005	<0.005	<0.005	0.053	<0.250	<0.005
RC07	mango	<0.005	<0.005	<0.005	<0.025	0.284	<0.005
RC08	lemon	<0.005	<0.005	<0.005	<0.025	0.720	<0.005
RC09	pineapple	<0.005	<0.005	<0.005	<0.025	0.745	<0.005
RC10	peach	<0.005	<0.005	<0.005	<0.025	0.256	<0.005
RC11	two apple	<0.005	<0.005	<0.005	0.149	0.295	<0.005
RC12	blueberry	<0.005	<0.005	<0.005	<0.025	0.259	0.021
RC13	apple	<0.005	<0.005	<0.005	<0.025	<0.250	<0.005
RC14	menthol	<0.005	<0.005	<0.005	<0.025	<0.250	<0.005
RC15	melon	<0.005	<0.005	<0.005	<0.025	0.414	<0.005
RC16	grape	<0.005	<0.005	<0.005	<0.025	0.276	<0.005
VPJ 1	melon	0.016	<0.005	<0.005	<0.025	0.382	0.100
VPJ 2	ice blueberry	0.015	<0.005	<0.005	0.097	0.423	0.069
VPJ 3	grape ice	0.068	<0.005	<0.005	<0.025	1.007	0.022
VPJ 4	double mango	0.025	<0.005	<0.005	<0.025	0.455	0.049
VPJ 5	kiwi dragon berry ice	0.023	<0.005	<0.005	<0.025	0.794	0.024
VPJ 6	banana ice	0.028	<0.005	<0.005	<0.025	0.586	0.045

Descriptive statistical analysis was applied to organize and interpret the distribution of inorganic element concentrations in the e-liquid samples. Normality tests indicated non-normal frequency distributions for all evaluated elements. The Mann–Whitney U test revealed statistically significant differences (*p* < 0.05) in As, Cr, and Hg levels between nicotine-containing and nicotine-free samples, suggesting a direct association between nicotine presence and the concentrations of these elements (Figure 1). In contrast, Cd, Pb, and Ni exhibited concentrations near or below their respective LOQs in both groups, with no statistically significant differences.

**Figure 1:**
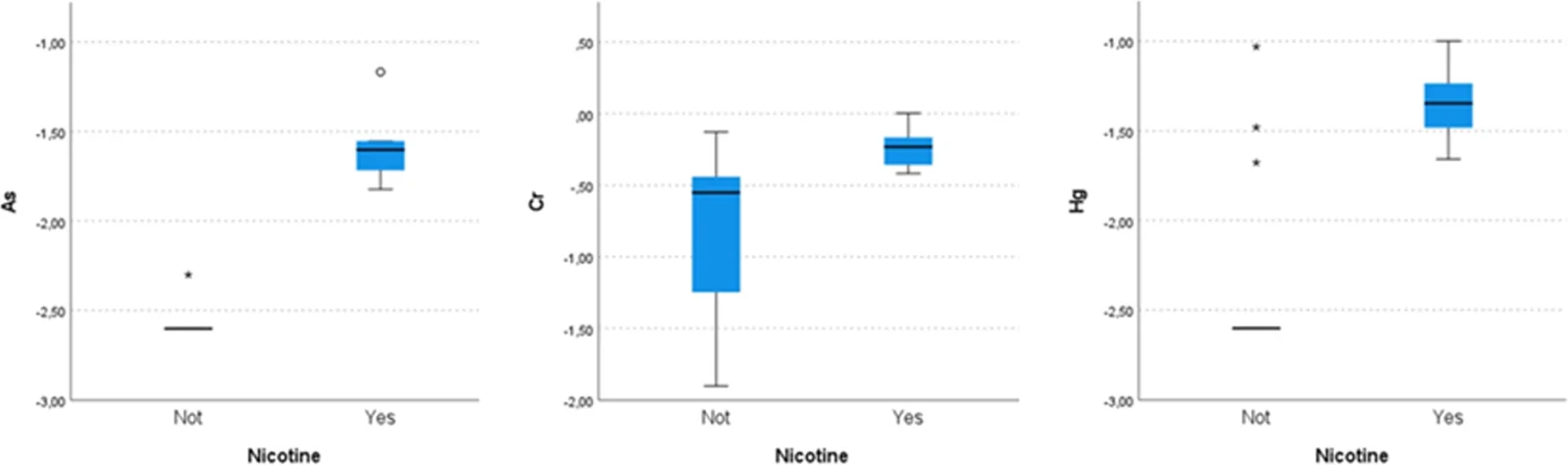
Results of the Mann–Whitney U test results for As, Cr, and Hg concentrations in nicotine-containing versus nicotine-free e-liquid samples.

The Kruskal–Wallis test was applied to evaluate whether flavor influenced the elemental composition, revealing no statistically significant differences among the flavors (*p* > 0.05). This finding suggests that, with respect to the trace elements investigated, flavoring does not appear to be a determining factor in chemical contamination. Spearman’s correlation showed strong positive correlations between As and both Cr and Hg, suggesting shared contamination pathways or comparable chemical behaviors during manufacturing or raw material sourcing.

Cluster analysis (CA) resulted in the formation of two main groups: (1) Cd, Pb, As, Hg, and Ni and (2) Cr. Group 1 was further subdivided into (1.1) Cd–Pb–Ni and (1.2) As–Hg subclusters (Figure 2), reflecting distinct origins and/or geochemical behaviors of the elements. Cd, Pb, and Ni are typically associated with metallic components and alloys, whereas As and Hg are characterized by greater volatility and adsorption. Together, these results highlight the influence of both chemical properties and production-related factors on the elemental distribution.

**Figure 2:**
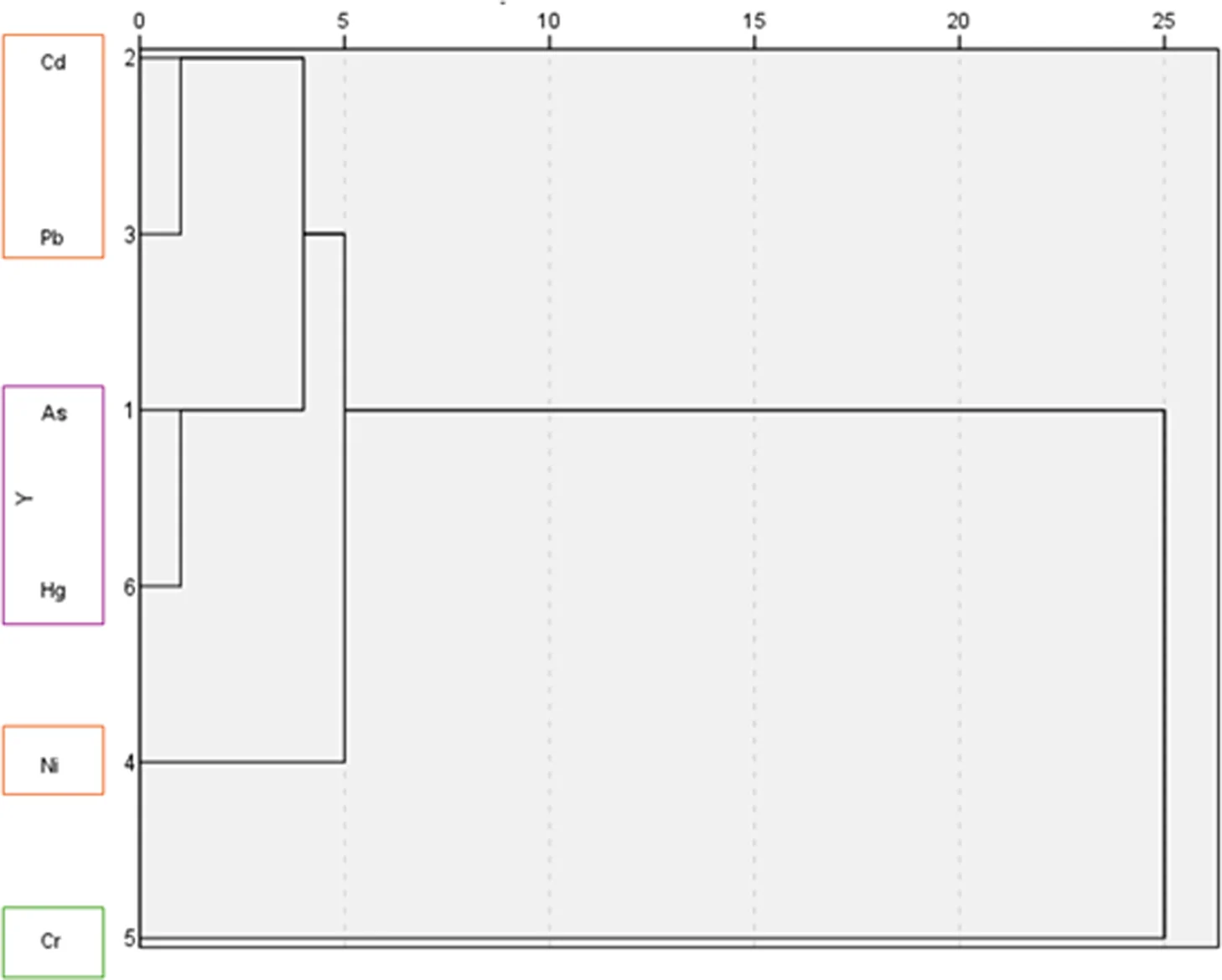
Cluster analysis (CA) dendrogram illustrating similarity patterns among inorganic elements detected in e-liquid samples.

### Nicotine Concentration

Nicotine concentrations in the analyzed e-liquids ranged from below the limit of detection (LOD) to 33.4 mg·mL^–1^ (Table 2). No nicotine was detected in products labeled as nicotine-free or containing low nicotine levels (RC04 and VPJ1). Among nicotine-containing products, measured concentrations closely matched the declared values for samples VPJ2 (14.7 mg·mL^–1^) and VPJ5 (32 mg·mL^–1^), which were consistent with the labeled concentrations of 15 mg·mL^–1^ and 35 mg·mL^–1^, respectively. By contrast, higher-than-declared concentrations were observed in both samples labeled as containing 20 mg·mL^–1^ of nicotine (VPJ3 and VPJ6). Only VPJ4 showed a lower-than-declared concentration, possibly due to nicotine degradation or conversion into other compounds. Discrepancies between labeled and measured nicotine concentrations are not uncommon and indicate insufficient quality control, potentially resulting in unintended user exposure to elevated nicotine doses.^18^ In the European Union, Canada, and the United Kingdom, the maximum permissible nicotine concentration in e-liquids is 20 mg·mL^–1^. In this study, the values of three samples exceeded this regulatory limit.^19^

**Table 2:** Declared and Measured Nicotine Concentrations in the Analyzed e-Liquids

sample Id	declared nicotine value (mg·mL^–1^)	measured nicotine value (mg·mL^–1^)
RC 04	0	<LOD^a^
VPJ 1	3	<LOD^a^
VPJ 2	15	14.7
VPJ 3	20	33.4
VPJ 4	20	5.3
VPJ 5	35	32.0
VPJ 6	20	31.6

a Limit of detection (LOD) of the analytical technique = 5.0 × 10^–4^ mg·mL^–1^.

### Analysis of Exposure to Inorganic Contaminants

To estimate the potential inhalation exposure, the highest concentrations of inorganic elements were converted to μg·mL^–1^ of e-liquid, using the experimentally determined density of each individual e-liquid sample (Table S3). Daily exposure was calculated for three consumption scenarios: light user (5 mL), moderate user (10 mL), and heavy user (20 mL).^20^ The PDE limits for inhalation established by the ICH were used as reference thresholds. The estimated daily exposure values for each element and user profile are presented in Table 3.

**Table 3:** Estimated Daily Exposure to As, Cd, Pb, Ni, Cr and Hg During ENDS Use for Different e-Liquid Consumption Profiles

element	PDE (μg·day^–1^)	light user (μg·day^–1^)	moderate user (μg·day^–1^)	heavy user (μg·day^–1^)
**As**	1.9	0.37	0.74	1.49
**Cd**	3.4	0.03^a^	0.06^a^	0.12^a^
**Pb**	5.0	0.30	0.59	1.18
**Ni**	6.0	0.84	1.67	3.35
**Cr**	2.9	5.51	11.03	22.06
**Hg**	1.2	0.56	1.13	2.26

a Calculated using the Limit of quantification (LOQ) of the element = 5.0 × 10^–3^ μg·g^–1^.

Two elements exhibited exposure levels exceeding the established PDE limits: Cr exceeded the inhalation PDE across all calculated exposure profiles, whereas Hg surpassed the limit under the heavy-user scenario. Cr occurs naturally in trivalent (Cr^3+^) and hexavalent (Cr^6+^) forms, with Cr^6+^ being considerably more toxic and classified as a human carcinogen. The ICP–MS method quantified total Cr and did not differentiate between Cr^3+^ and Cr^6+^. Therefore, the risk assessment was based on the inhalation PDE established for Cr^3+^, which has been reported as the predominant chromium species in e-liquids and aerosols.^21^ Although Cr^3+^ is considerably less toxic than Cr^6+^, inhalation exposure to Cr^3+^ has been associated with airway toxicity and adverse respiratory effects.^22^ Hg and its compounds are highly toxic systemically and act as respiratory tract irritants capable of exacerbating asthma symptoms.^14^ Chronic inhalation of low concentrations of mercury vapor may result in neurological impairment, memory loss, skin rashes, and kidney failure.^23^

Despite these findings, estimating daily exposure in e-cigarette studies remains challenging and should be interpreted with caution, given the high variability in ENDS usage patterns. This variability stems from differences in device characteristics, e-liquid formulations, and the behavior of users. Moreover, many users cannot accurately report their device specifications or consumption habits.^24^ Studies also indicate that aerosols generated by ENDS are chemically more complex than the original e-liquids because of thermal degradation and vaporization processes. Therefore, inorganic contaminants may be present at higher concentrations in inhaled aerosols as a result of leaching from heating elements and other device components.^16,25^

## Conclusion

Several e-liquid samples contained measurable concentrations of As, Pb, Ni, Cr, and Hg, with nicotine significantly influencing the concentrations of As, Cr, and Hg. These findings indicate that ENDS may serve as relevant sources of exposure to toxic elements, particularly Cr and Hg, as the estimated daily inhalation exposures exceeded the PDE limits established by the ICH. In addition, substantial variability and discrepancies between the measured and labeled nicotine concentrations were observed, highlighting the pronounced compositional heterogeneity among e-liquids, especially those available on the illicit market. Collectively, these results underscore the need for specific regulatory guidelines addressing the toxicological evaluation of ENDS, as well as standardized usage parameters that enable more realistic human exposure assessments. Future research should incorporate a larger number of samples, evaluate a broader range of chemical contaminants, and include analyses of the aerosols generated during use to achieve a more comprehensive toxicological profile and strengthen the evidence on the health risks associated with electronic cigarette use.

## Supplementary Material



## Notes

Supporting Information tables containing the ICP–MS operating conditions, method validation parameters for the determination of inorganic elements in e-liquids, and the experimentally determined densities of the analyzed e-liquid samples (PDF)
