# Supplementary material for: Exposure to Toxicologically Relevant Inorganic Elements and Nicotine Content Variability in Electronic Nicotine Delivery System (ENDS) E‑Liquids
Source: Chem Res Toxicol. 2026 Aug 31;39(9):1847–53. doi: 10.1021/acs.chemrestox.6c00266 (PMC13599469; doi:10.1021/acs.chemrestox.6c00266)
Supplement: Supplementary file 1 [file tx6c00266_si_001.pdf]

Supporting Information

Exposure to Toxicologically Relevant Inorganic Elements and Nicotine Content Variability in Electronic Nicotine Delivery System (ENDS) E-liquids

Lara M. Catarino[†]*, Giovanna M. Iozzi*[†]*, Cristiane B. Silva*[†]*, Santos A.V. Neto*[†]*, Graziele A. Reis*[‡]*, Silvana C. Jacob*[†]*, and Lisia M. G. dos Santos*[†] [*].

[†] Inorganic Elements Sector, Department of Chemistry, National Institute for Quality Control in Health (INCQS), Oswaldo Cruz Foundation (Fiocruz), Rio de Janeiro, RJ, 21040-900, Brazil.

[‡] State University of Rio de Janeiro (UERJ), Maracanã, Rio de Janeiro, RJ, 20550-013, Brazil.

Table of Contents

Table S1. ICP-MS operating conditions...S2

Table S2. Validation parameters of the ICP-MS method for the determination of inorganic elements in e-liquids...S3

Table S3. Experimentally determined density values of the analyzed e-liquid samples...S4

Table S1. ICP-MS operating conditions.

ICP-MS Parameter	Value
**ICP RF power**	1600W
**Plasma gas flow**	17.5 L·min^{-1}
**Auxiliary gas flow**	1.4 L·min^{-1}
**Nebulizer gas flow**	0.84 L·min-^{1}
**Dwell time**	15 ms
**Replicates**	3
**Sweeps per reading**	50
**Acquisition mode**	Standard (As, Cd, Hg, Ni, Pb); KED (Cr)
**Collision gas flow (He)**	1.25 L·min^{-1}

Table S2. Validation parameters of the ICP-MS method for the determination of inorganic elements in e-liquids.

Parameters	As	Cd	Pb	Ni	Cr	Hg
**LOD (µg·L^{-1})**	0.011	0.004	0.018	0.189	0.138	0.011
**Instrumental LOQ (µg·L^{-1})**	0.1	0.1	0.1	0.5	5.0	0.1
**Method LOQ (µg·g^{-1})**	0.005	0.005	0.005	0.025	0.250	0.005
**Recovery at LOQ[a] (%)**	98 - 102	79-82	95-106	92-101	105-111	83-97
**Recovery[a] (%)**	100 - 103	79 – 81	94 - 110	95 - 111	101 - 114	80 - 95
**Repeatability[b] (% RSD)**	0.4	0.9	1.5	4.3	2.2	5.0
**Intermediate precision[b] (% RSD)**	0.9	1.5	2.1	1.6	2.6	6.7
**Working range (µg·L^{-1})**	0.1 - 10.0	0.1 - 10.0	0.1 - 10.0	0.5 - 10.0	5.0 - 30.0	0.1 - 10.0

[a]**Acceptance criteria for accuracy:** Recovery acceptance ranges were 40–120% at the LOQ (0.1 and 0.5 µg·L^{-1}) and at the medium concentration level (1.0 µg·L^{-1}), and 60–115% at the high concentration level (10.0 µg·L^{-1}) for As, Cd, Pb, Ni, and Hg. For Cr, the acceptance range was 60–115% at the LOQ (5.0 µg·L^{-1}), medium (15.0 µg·L^{-1}), and high (30.0 µg·L^{-1}) concentration levels.

[b]**Acceptance criteria for precision:** Precision acceptance limits were ≤ 21% for repeatability and ≤ 32% for intermediate precision.

Table S3. Experimentally determined density values of the analyzed e-liquid samples.

Sample ID	Density ($g{\cdot}mL^{-1}$)
RC 01	1.146 ± 0.005
RC 02	1.119 ± 0.001
RC 03	1.155 ± 0.004
RC 04	1.160 ± 0.011
RC 05	1.151 ± 0.003
RC 06	1.146 ± 0.011
RC 07	1.150 ± 0.002
RC 08	1.152 ± 0.001
RC 09	1.141 ± 0.008
RC 10	1.146 ± 0.007
RC 11	1.123 ± 0.002
RC 12	1.148 ± 0.001
RC 13	1.155 ± 0.002
RC 14	1.135 ± 0.006
RC 15	1.111 ± 0.003
RC 16	1.104 ± 0.002
VPJ 1	1.129 ± 0.008
VPJ 2	1.264 ± 0.003
VPJ 3	1.095 ± 0.006
VPJ 4	1.206 ± 0.004
VPJ 5	1.139 ± 0.012
VPJ 6	1.119 ± 0.002

The density of each e-liquid sample was determined experimentally in triplicate at room temperature and is reported as the mean ± standard deviation (n = 3).
